# Soil heat extremes can outpace air temperature extremes

**DOI:** 10.1038/s41558-023-01812-3

**Published:** 2023-09-21

**Authors:** Almudena García-García, Francisco José Cuesta-Valero, Diego G. Miralles, Miguel D. Mahecha, Johannes Quaas, Markus Reichstein, Jakob Zscheischler, Jian Peng

**Affiliations:** 1 Department of Remote Sensing, Helmholtz Centre for Environmental Research—UFZ, Leipzig, Germany; 2 Remote Sensing Centre for Earth System Research, Leipzig University, Leipzig, Germany; 3 Hydro-Climate Extremes Lab (H-CEL), Ghent University, Ghent, Belgium; 4 Leipzig Institute for Meteorology, Leipzig University, Leipzig, Germany; 5 Department of Biogeochemical Integration, Max Planck Institute for Biogeochemistry, Jena, Germany; 6 Department of Computational Hydrosystems, Helmholtz Centre for Environmental Research—UFZ, Leipzig, Germany

**Keywords:** Atmospheric dynamics, Projection and prediction, Climate-change impacts

## Abstract

Quantifying changes in hot temperature extremes is key for developing adaptation strategies. Changes in hot extremes are often determined on the basis of air temperatures; however, hydrology and many biogeochemical processes are more sensitive to soil temperature. Here we show that soil hot extremes are increasing faster than air hot extremes by 0.7 °C per decade in intensity and twice as fast in frequency on average over Central Europe. Furthermore, we identify soil temperature as a key factor in the soil moisture–temperature feedback. During dry and warm conditions, the energy absorbed by the soil is used to warm the soil, increasing the release of sensible heat flux and surface air temperatures. This increase in surface air temperature leads to a higher atmospheric demand for water, increasing soil evaporation, which may further dry and warm the soil highlighting the contribution of soil moisture–temperature feedback to the evolution of hot extremes in a warming climate.

## Abstract (web summary)

Changes in air temperature are usually considered for quantifying changes in temperature extremes such as heatwaves. This study shows that the incidence of heat extremes in soils is increasing faster than air temperature in some regions, with implications for hydrological and biogeochemical processes.

## Main

Extreme temperatures and associated disasters, such as crop loss, wildfires, water scarcity, air pollution and CO_2_ release from ecosystems, exert a heavy toll on society and ecosystems^1,2^. For example, a death toll of 55,000, more than 1 million ha of burned land, and US$15 billion of total economic loss were associated with the 2010 Russian heatwave^1^. A recent study has also related more than 100,000 deaths from 2002 to 2015 to extreme temperatures in Latin American cities^3^. More generally, there is evidence of a positive trend in the intensity and frequency of extreme temperatures at global and regional scales that is increasing public concern^4^.

Despite the negative consequences of heatwaves and the observed trends in their occurrence and evolution, understanding of how climate change may affect them is still limited^5^. This is in part caused by the incomplete knowledge of processes controlling the evolution of heat extremes, such as land–atmosphere coupling. This knowledge gap is illustrated, for example, in the different levels of land–atmosphere coupling represented in climate models^6,7^. Land conditions can intensify and propagate heatwaves via diabatic heating^8^, in the worst case leading to mega-heatwaves^9^. In the presence of persistent high-pressure systems, soil moisture deficits and their induced reduction in evaporation may lead to the warming of the land and a larger fraction of net radiation dissipated as sensible heat into the atmosphere^10^. This directly contributes to the development or intensification of local heatwaves as measured by surface air temperatures, and may further increase soil desiccation^11^ and extreme temperatures^9^ in downwind areas. Therefore, the observed and projected changes in soil conditions under climate change have been proposed as an important driver of future changes in the intensity and frequency of heatwaves via shifts in the energy partition at the land surface^10^.

Although soil temperature links soil moisture and air temperature and is one of the main drivers of the terrestrial carbon cycle, the study of the more direct role of soil temperature in land–atmosphere feedbacks has not received as much attention as the role of soil moisture dynamics^5,10^. This is partially due to the tight coupling between air and soil temperatures at climate temporal scales. However, the relationship between air and soil temperatures is largely influenced by changes in land cover, aerodynamic conductance, soil water content and associated changes in soil properties^12^. While trends vary across meteorological stations, higher warming rates in soil than air have been recorded in China^13^ and Germany^14^, with meteorological stations in China also reporting different trends in maximum soil and air temperatures^15^.

In this Article, we explore the evolution of soil hot extremes over Europe during the past decades, and compare this evolution with that of hot extremes based on near-surface air temperatures. We provide evidence of regional differences between changes in soil and air hot extremes based on meteorological observations (FLUXNET2015 dataset^16^, the Integrated Carbon Observation System network (ICOS)^17^, the Agenzia Regionale per la Prevenzione e Protezione Ambientale del Veneto (ARPAV)^18^, Deutscher Wetterdienst (DWD)^19^ and Météo France^20^), remote sensing data^21^ and the ERA5Land re-analysis products^22^. Furthermore, simulations from the Earth system models (ESMs) participating in the sixth phase of the Coupled Model Intercomparison Project Phase 6 (CMIP6) (ref. ^23^) are used to investigate the role of soil temperature in the evolution of near-surface extreme temperatures in a warming climate.

## Soil hot extremes in the recent past

**Fig. 1:**
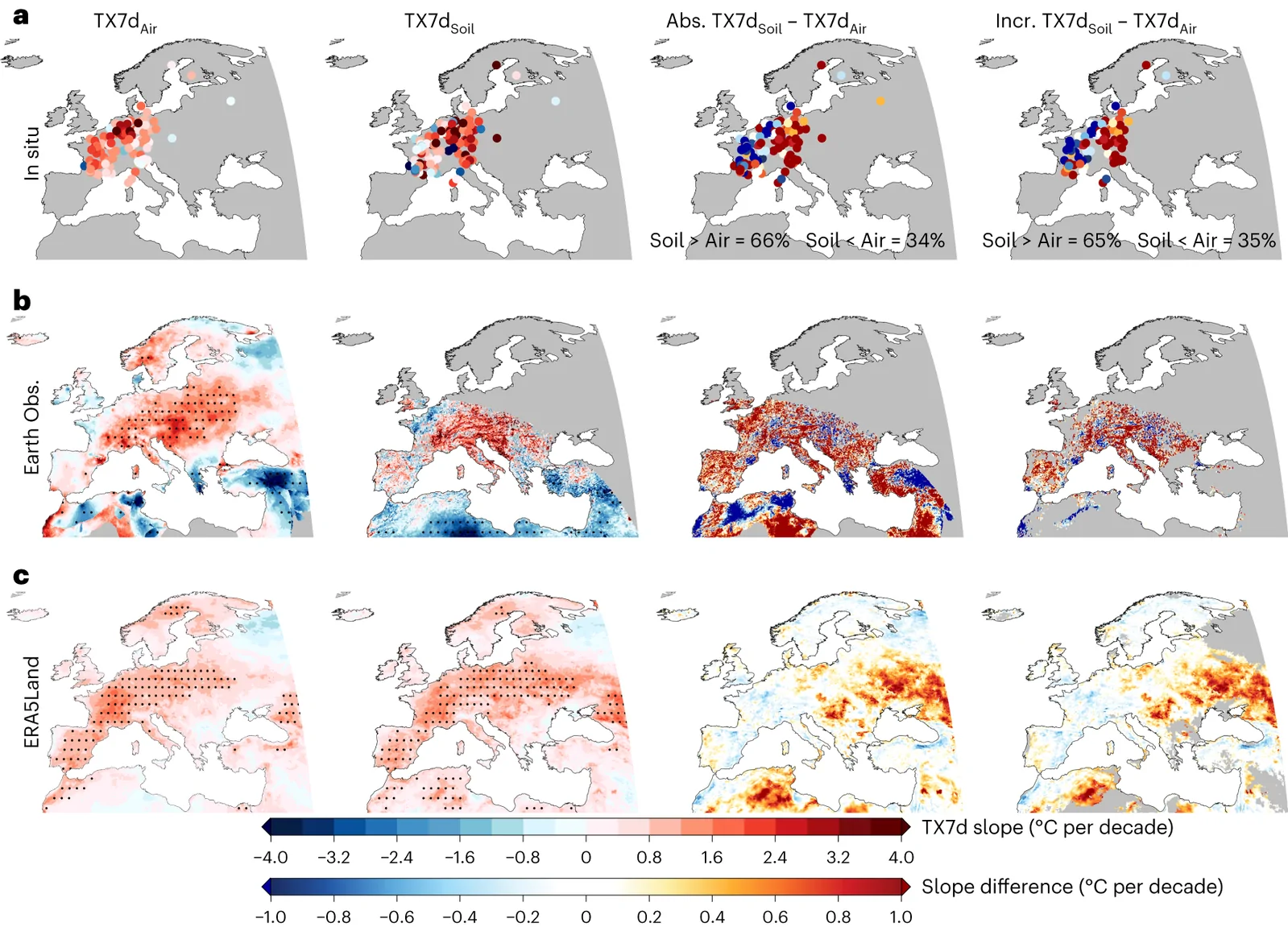
TX7d trends based on air and soil temperatures from 1996 to 2021 over Europe. **a**–**c**, From left to right, trend in TX7d based on air (TX7d_Air_) and soil temperatures (TX7d_Soil_), the difference between absolute values of trends in soil and air (Abs. TX7d_Soil_ − TX7d_Air_), and the difference between trends where both trends are positive (Incr. TX7d_Soil_ − TX7d_Air_). The TX7d index is defined as the mean value of daily maximum temperatures over the hottest week per year. Results are obtained from meteorological stations (**a**), a combination of CM SAF satellite data and Earth observations (E-OBS gridded dataset) (**b**) and the ERA5Land re-analysis (**c**). Dots indicate areas with significant trends above the 90% confidence level.

**Extended Data Fig. 1:**
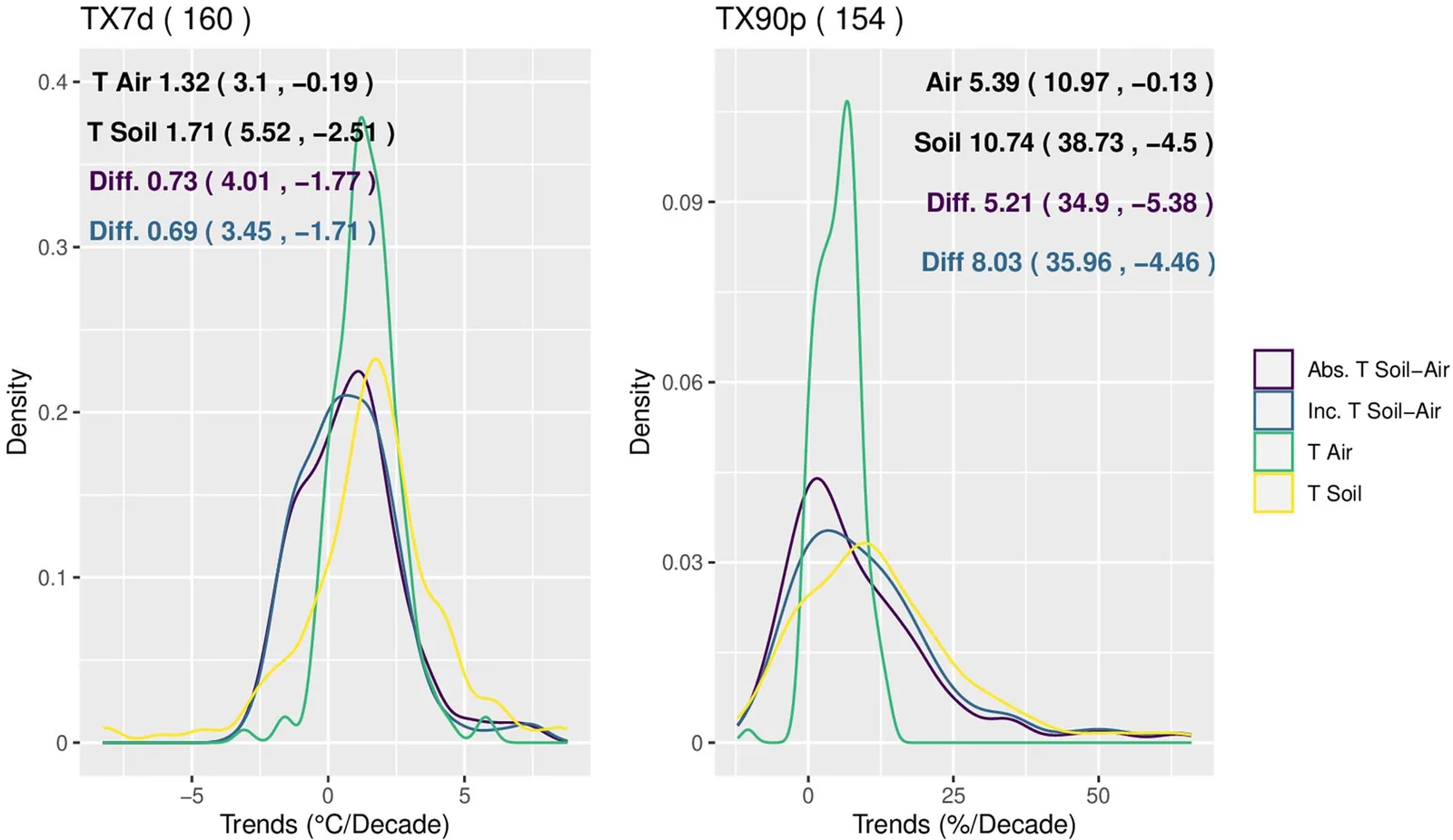
Probability distributions of trends in TX7d and TX90p from in-situ data. Probability distributions of TX7d and TX90p trends based on air (T Air) and soil (T Soil) observations, the difference of the absolute values of trends in soil and in air (Abs. T Soil - Air) and the difference between soil and air trends at stations with positive trends (Inc. T Soil - Air). Numbers indicate the median (95th and 5th percentiles) of the distributions based on air and soil temperatures and the differences between soil and air slopes.

Trends in the intensity of hot extremes based on air and soil temperatures are investigated using the annual TX7d index defined as the mean of daily maximum temperatures during the hottest week per year. The TX7d trends based on air and soil temperatures from meteorological stations, remote sensing products and re-analysis products show positive trends in air and soil TX7d index from 1996 to 2021 in Central Europe (Fig. 1). Trends in soil TX7d show larger spatial variability than in air based on data from meteorological observations (Extended Data Fig. 1), which indicates that local processes and soil heterogeneity have a stronger impact on soil temperature extremes than in air (Fig. 1a). The comparison of TX7d trends in soil and air shows 66% of measurements with larger absolute values of trends in soil than in air, that is, soil extremes are changing faster than air extremes at those stations. Similar percentages are obtained when only observations with positive trends are compared, that is, 65% of measurements indicate that TX7d trends in soil are increasing faster than for air temperature. Most stations that portray a faster increase in soil than in air temperature extremes are located over Germany, Italy and southern France, while the opposite case is found over central and northern France, Belgium and the Netherlands.

Results from the combination of the E-OBS gridded product^24^ and the CM SAF remote sensing data^21^ also support the existence of faster TX7d trends in soil than in air (Fig. 1b). A higher TX7d increase based on air temperatures than on skin temperatures is observed over some forested areas in north-eastern Spain, north-western Italy, south-western Germany and Greece (Fig. 1b). This is related to the fact that over forested areas the satellite estimates do not correspond to soil temperatures but to canopy temperatures at heights much higher than the topsoil.

**Extended Data Fig. 2:**
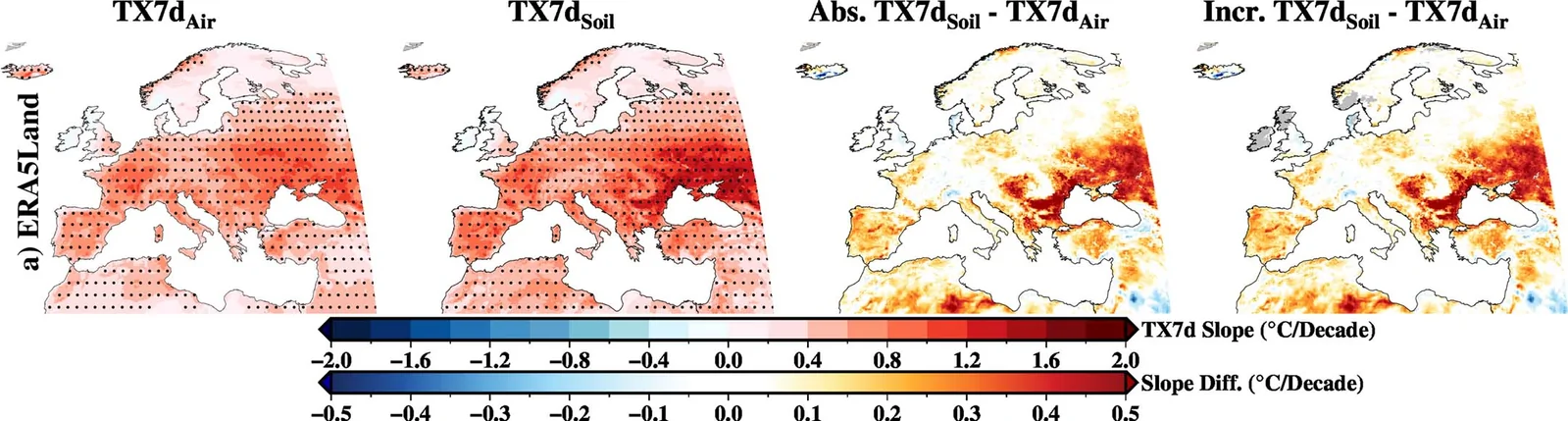
TX7d trends based on air and soil temperatures from 1970 to 2021 over Europe based on the ERA5Land reanalysis product. From left to right, trend in TX7d based on air temperatures, trend in TX7d based on soil temperatures, the difference between absolute values of trends in soil and air, and the difference between trends in soil and in air where both trends are positive. Dots indicate areas with significant trends above the 90% confidence level.

Regions with faster TX7d trends in soil than in air are also found on the basis of the ERA5Land data, which relies on a modelling framework to estimate soil temperature (Fig. 1c). In this dataset, the spatial patterns of TX7d trends in soil and air are very similar, with values much closer than for the observational products. The ERA5land product shows slightly faster increase in air than in soil extreme temperatures over France and the opposite behaviour over eastern Germany and western Poland. Larger differences are found over central-eastern Europe, where TX7d in soil increases faster than in air by more than 0.5 °C per decade (Fig. 1c). The analysis of ERA5Land outputs for a longer period 1970–2021 yields similar conclusions (Extended Data Fig. 2), which indicates that the length of the selected period is not affecting the conclusions of this study.

**Extended Data Fig. 3:**
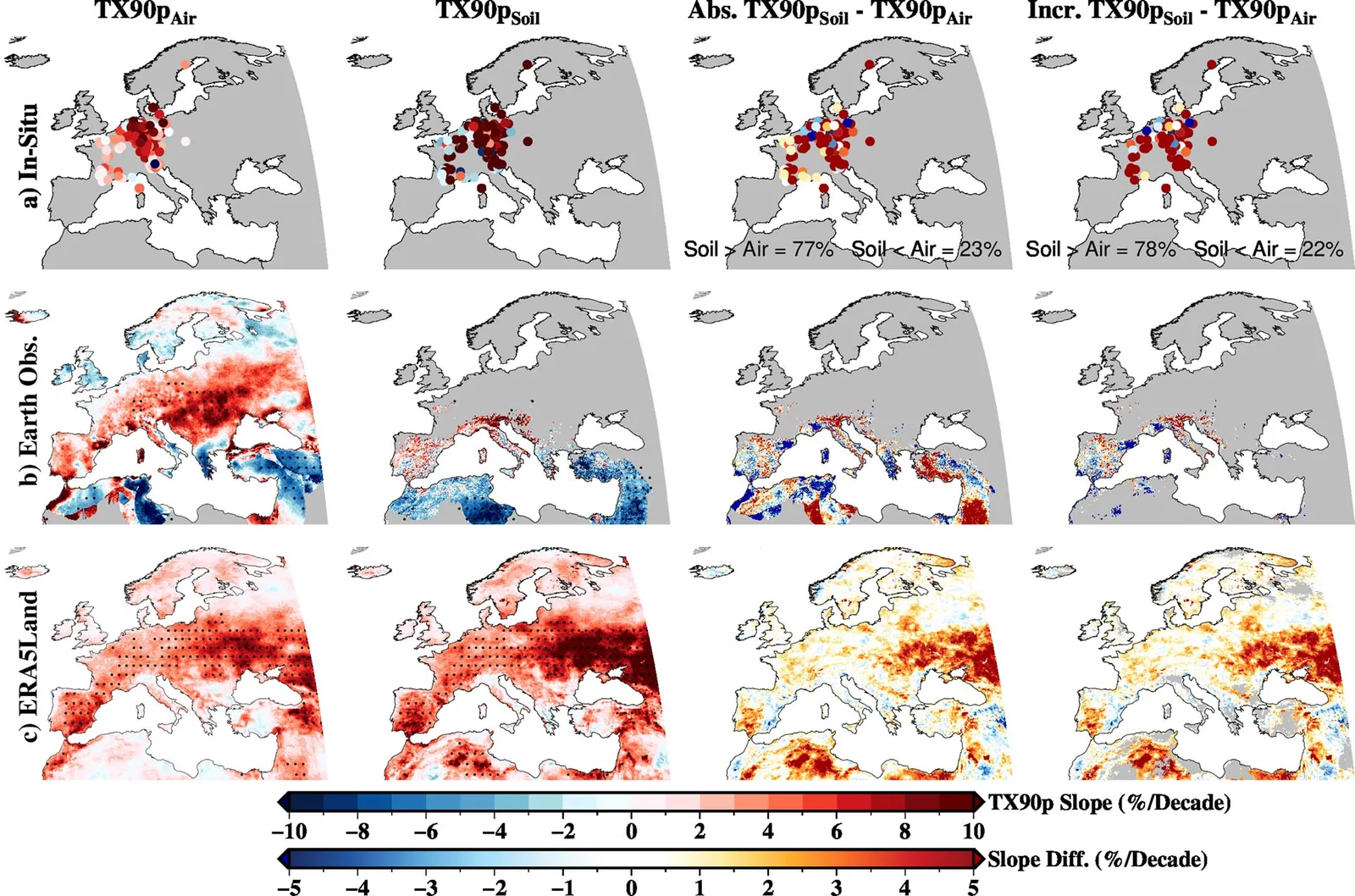
TX90p trends based on air and soil temperatures in summer from 1996 to 2021 over Europe. From left to right, trend in TX90p based on air (TX90p_Air_) and soil temperatures (TX90p_Soil_), the difference between absolute values of trends in soil and air (Abs. TX90p_Soil_ - TX90p_Air_), and the difference between trends where both trends are positive (Incr. TX90p_Soil_ - TX90p_Air_). TX90p is defined as the percentage of hot days in boreal summer per year. Results are obtained from meteorological stations (a), a combination of CM SAF satellite data and the E-OBS dataset (b) and the ERA5Land reanalysis (c). Dots indicate areas with significant trends above the 90% confidence level.

**Extended Data Fig. 4:**
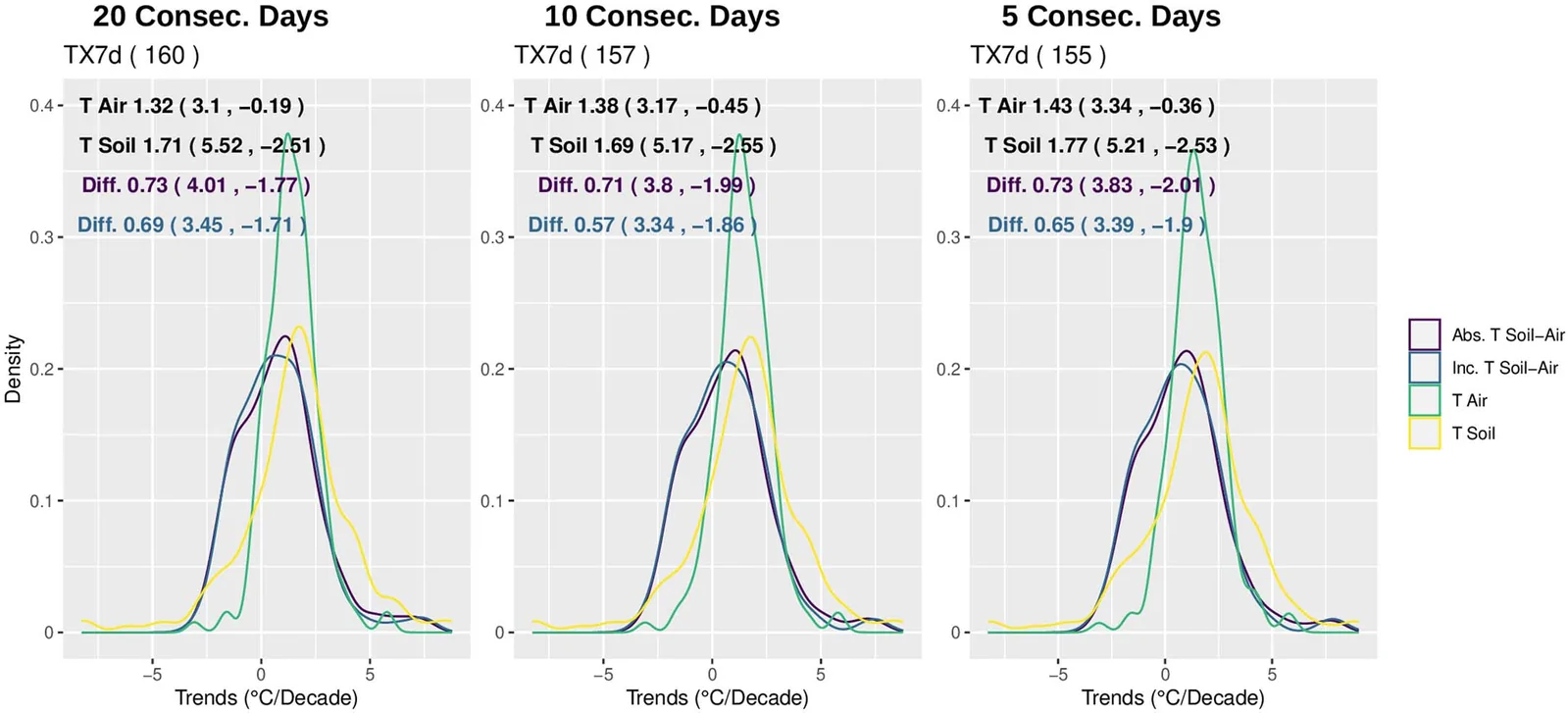
Probability distribution of trends in TX7d from in-situ data using different criteria for the missing values. Each panel shows trends in TX7d based on air (T Air) and soil (T Soil) observations, the difference of the absolute values of trends in soil and in air (Abs. T Soil - Air) and the difference between soil and air trends at stations with positive trends (Inc. T Soil - Air). Numbers indicate the median (95th and 5th percentiles) of the distributions based on air and soil temperatures and the differences between soil and air slopes.

**Extended Data Fig. 5:**
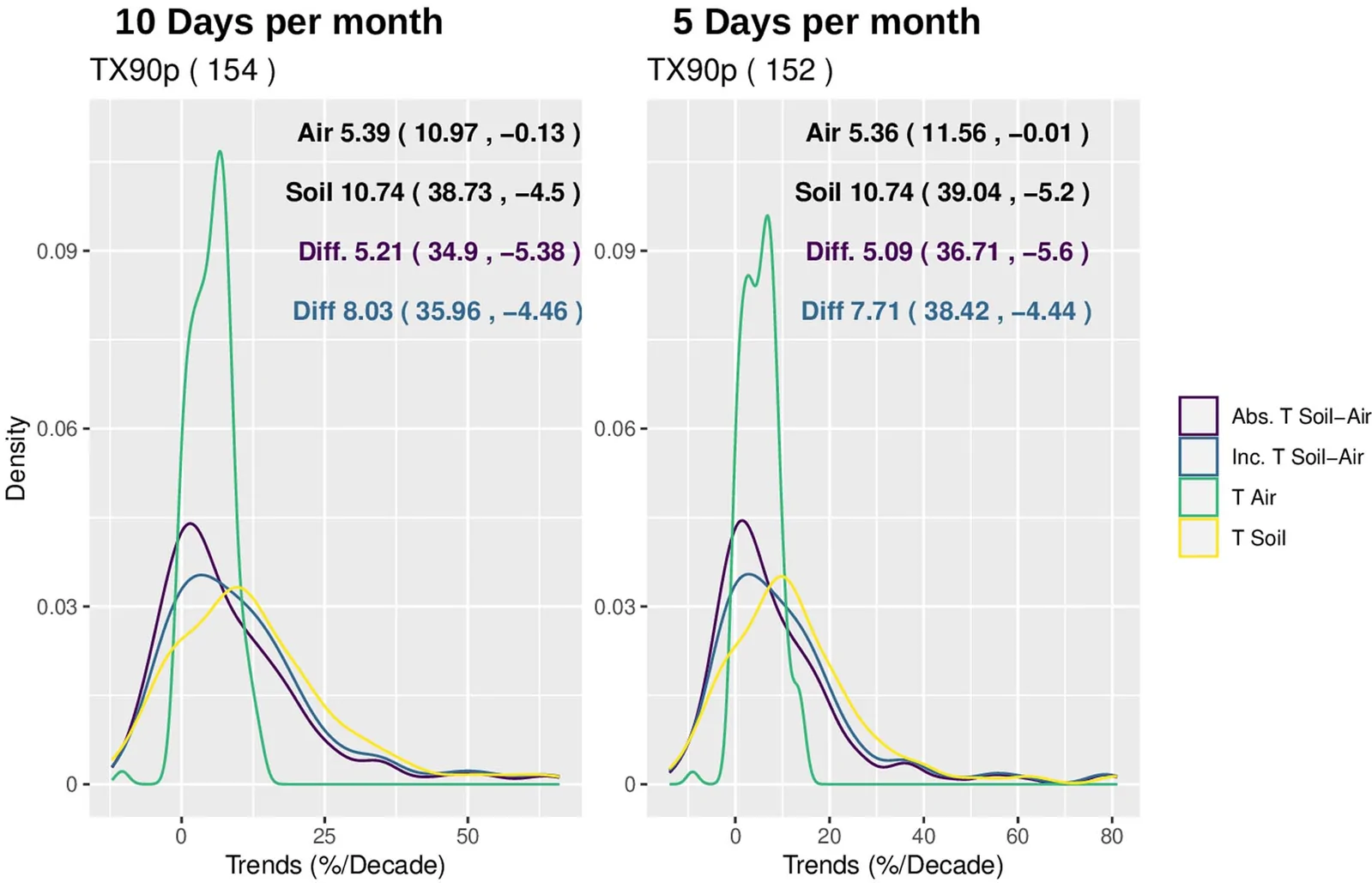
Probability distribution of trends in TX90p from in-situ data using different criteria for the missing values. Each panel shows trends in TX90p based on air (T Air) and soil (T Soil) observations, the difference of the absolute values of trends in soil and in air (Abs. T Soil - Air) and the difference between soil and air trends at stations with positive trends (Inc. T Soil - Air). Numbers indicate the median (95th and 5th percentiles) of the distributions based on air and soil temperatures and the differences between soil and air slopes.

Hot extremes are also increasing in frequency using air and soil temperatures over Central Europe (Extended Data Fig. 3). Soil and air differences are consistent and even more robust when using a widely used frequency index for hot extremes; the summer mean of the monthly TX90p index defined as the percentage of days per month when daily maximum temperature is higher than a statistical threshold. In this study, the threshold was estimated as the 90th percentile of daily maximum temperatures for the first 10 years of the period to be able to use the in situ observations (for more details, see Methods). Different criteria for managing missing values were tested for the TX7d and TX90p indices, reaching similar results and conclusions (Extended Data Figs. 4 and 5). This indicates that the rapid increase in soil heat extremes in comparison with air heat extremes is independent of the index definition but differences between slopes in soil and air are reliant on the resolution and local characteristics of the data source. Thus, soil hot extremes are increasing 0.7 °C per decade faster than air hot extremes in intensity and twice as fast as air hot extremes in frequency on average over Central Europe based on the station data (Extended Data Fig. 1).

**Extended Data Fig. 6:**
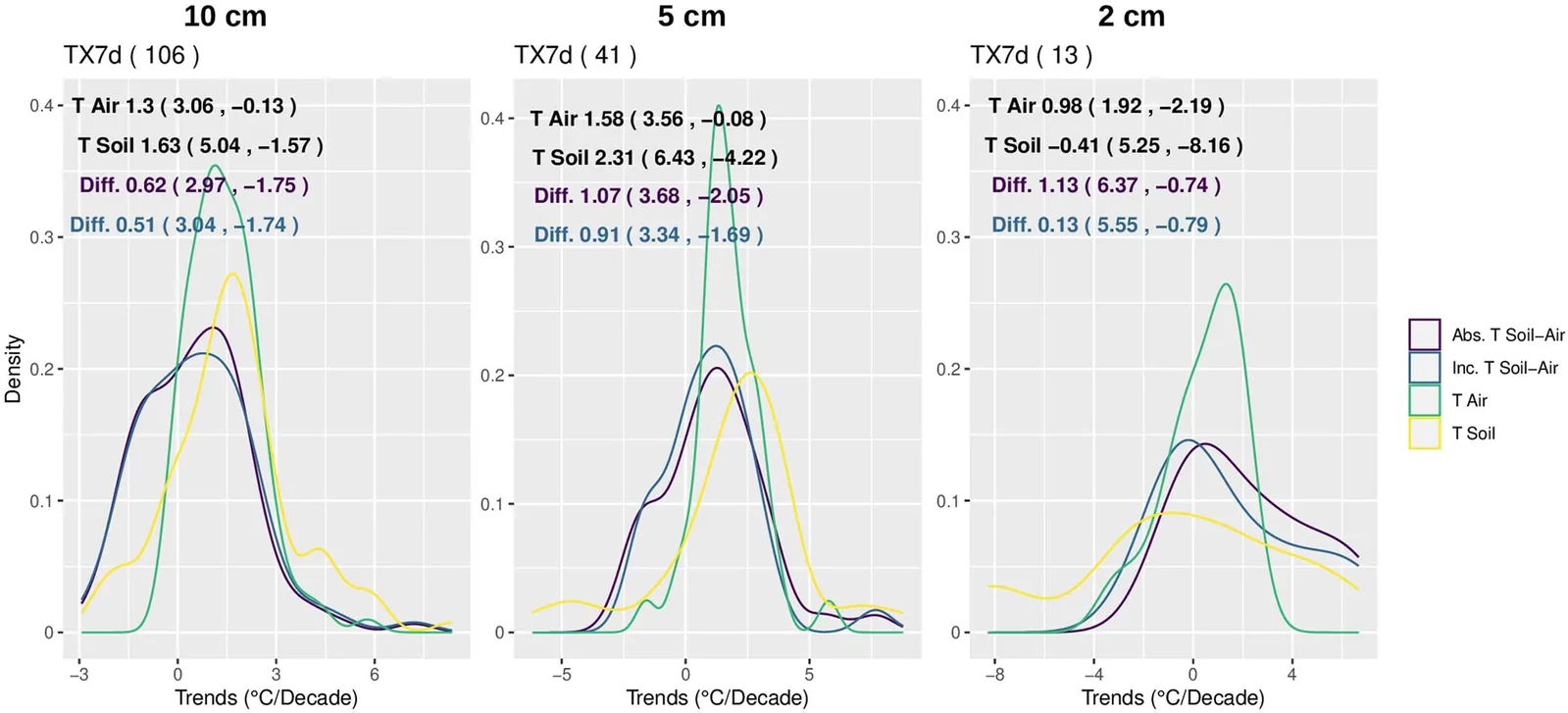
Probability distributions of trends in TX7d from in-situ data classified by soil layer (10 cm, 5 cm and 2 cm). Each panel shows trends in TX7d based on air (T Air) and soil (T Soil) observations classified by soil temperature depth, the difference of the absolute values of trends in soil and in air (Abs. T Soil - Air) and the difference between soil and air trends at stations with positive trends (Inc. T Soil - Air). Numbers indicate the median (95th and 5th percentiles) of the distributions based on air and soil temperatures and the differences between soil and air slopes.

Regarding the spatial differences between trends in air and soil hot extremes (Fig. 1 and Extended Data Table 1), the literature has identified the role of surface soil moisture in the thermal coupling between air and soil temperatures during summer^7,12^. However, there are many factors that affect soil moisture and show large spatial heterogeneity, such as land cover and soil composition. For example, land cover alters the air and soil thermal coupling due to different surface roughness and root zone depth. Vegetation with deep roots may have access to groundwater, reducing the limitations in evaporation associated with low water content in the shallow soil layers (horizons). By contrast, crops or grasslands with shallow roots will only access shallow groundwater, reducing transpiration. Additionally, water infiltration into the soil is driven by soil texture^25^ and can be affected by plant species richness^26^. Thus, all these processes have an effect on the relationship between air and soil temperatures, leading to the spatial differences in Fig. 1. Soil depth may have an important role in the evolution of soil hot extremes (Supplementary Information). However, due to the different number of stations included in this study, providing data at each soil layer (up to 10 cm) and the high spatial variability of our results, we do not find a clear relationship between depth and trends in soil hot extremes (Extended Data Fig. 6).

## Soil temperature in the soil moisture–temperature feedback

**Fig. 2:**
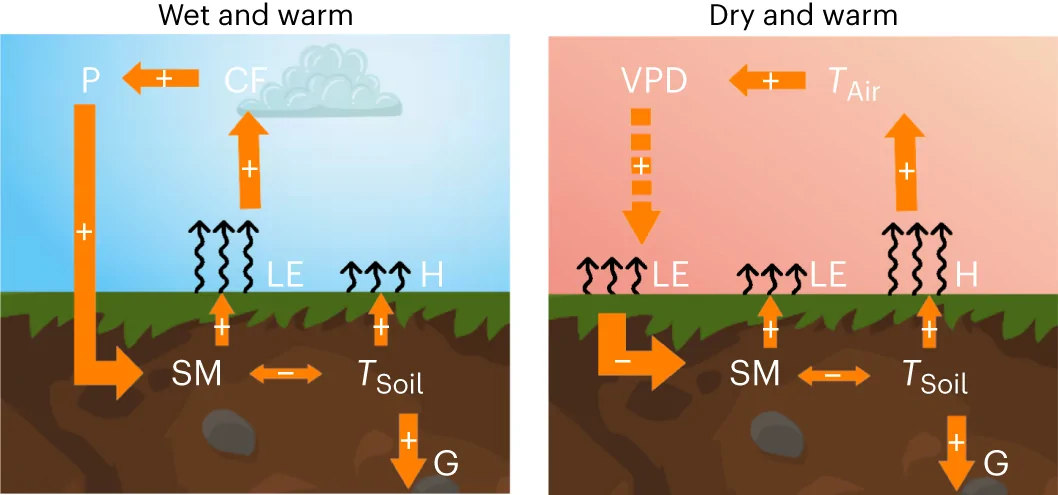
Soil temperature in the soil moisture–temperature feedback. During wet and warm conditions, net radiation is mainly released from the soil as latent heat (LE). This process decreases soil temperatures (*T*_Soil_) and increases cloud fraction (CF), leading to higher precipitations (P), which may further increase soil moisture (SM). During dry and warm conditions, net radiation is mainly used to raise *T*_Soil_, increasing sensible (H) and ground (G) heat fluxes, while LE is constrained by SM. The release of H into the atmosphere increases local and/or remote air temperatures (*T*_Air_), leading to vapour pressure deficits (VPDs). High VPD increases the demand for LE, further drying out and warming the soil. + (−) symbols indicate a direct (inverse) relationship, while arrows indicate the direction of the feedback.

**Extended Data Fig. 7:**
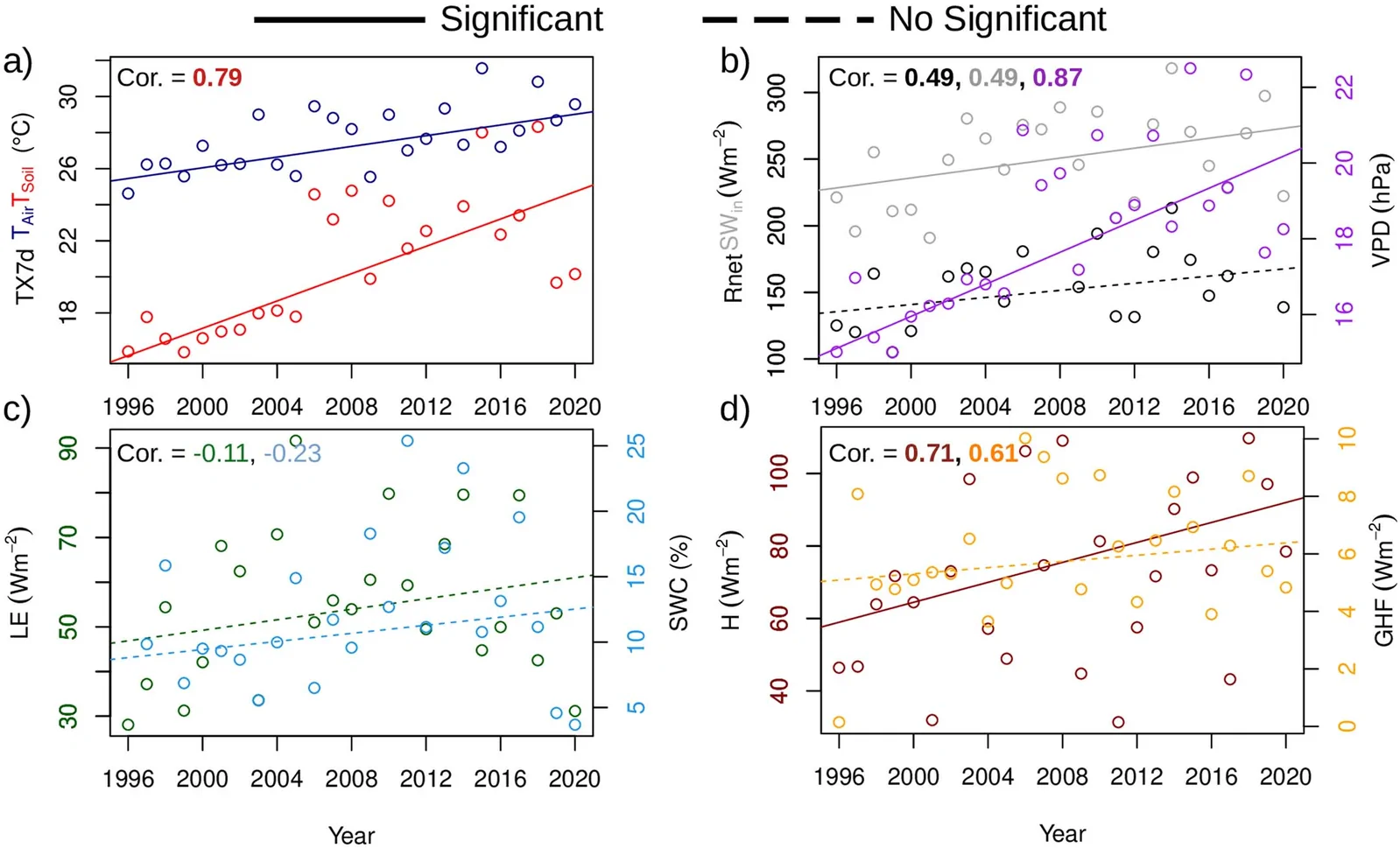
Trends at DE-Tha station during the hottest week per year from 1996 to 2020. Each panel shows trends in TX90p based on air (T Air) and soil (T Soil) observations, the difference of the absolute values of trends in soil and in air (Abs. T Soil - Air) and the difference between soil and air trends at stations with positive trends (Inc. T Soil - Air). Numbers indicate the median (95th and 5th percentiles) of the distributions based on air and soil temperatures and the differences between soil and air slopes.Trends in a) TX7d index based on air and soil (2 cm) temperatures; b) net radiation (Rnet), incoming shortwave radiation (SWin) and vapour pressure deficit (VPD); c) latent heat flux (LE) and soil water content (2 cm) (SWC); d) sensible (H) and ground (GHF) heat flux. The hottest week per year for all variables was identified using the TX7d index based on air temperatures. Significant trends above the 90% confidence level are represented with solid lines. Numbers indicate the Pearson’s correlation coefficient of each variable with the TX7d index based on air temperatures. Bold numbers indicates significance above the 90% confidence level.

**Extended Data Fig. 8:**
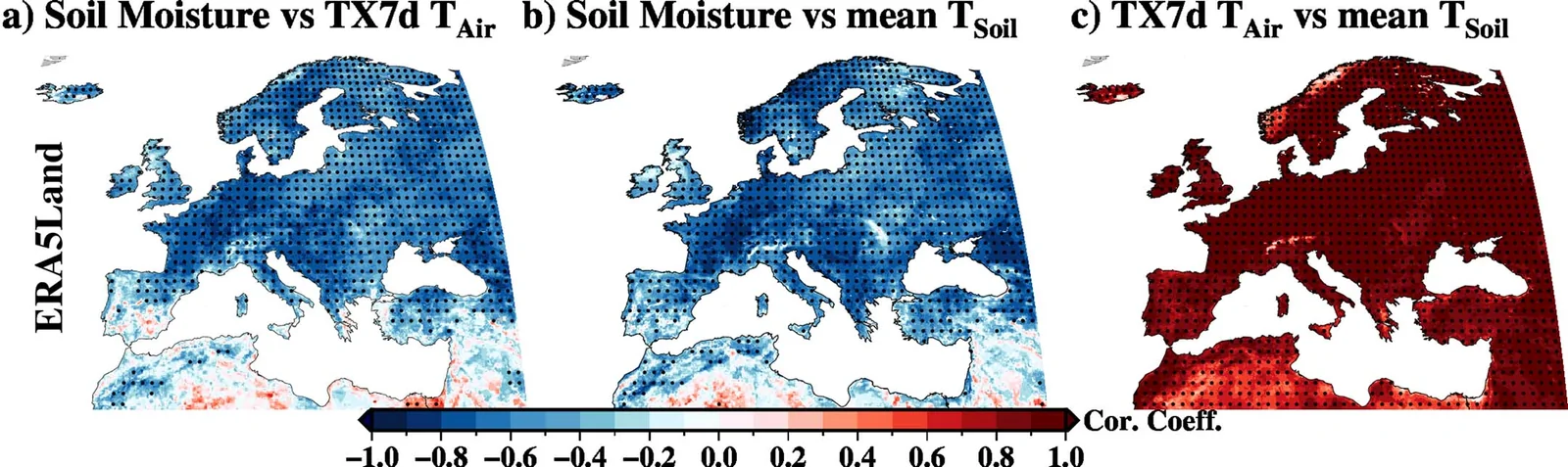
TX7d trends based on air and soil temperatures from 1970 to 2021 over Europe based on the ERA5Land reanalysis product. a) Pearson’s correlation coefficients between mean soil moisture and the TX7d index based on air temperatures. b) Pearson’s correlation coefficients between mean soil moisture and mean soil temperature. c) Pearson’s correlation coefficients between mean soil temperature and the TX7d index based on air temperatures. All correlation coefficients were estimated based on the ERA5Land data, using the mean of each variable during the hottest week per year identified by the TX7d_Air_ index from 1996 to 2020. Dots correspond with significance above the 90% confidence level.

The evolution of above surface hot extremes is particularly sensible to land–atmosphere interactions over dry or transitional areas during sunny anticyclonic conditions^27^. During wet and warm conditions, net radiation is mainly released from the soil in the form of latent heat, cooling soil temperatures and limiting sensible heat flux. The release of latent heat from the soil increases atmospheric water content, leading to higher precipitation rates, which are usually associated with increases in soil water content. In contrast, net radiation is mainly used to raise soil temperature during dry and warm conditions due to soil water limitations. If soil temperature is then higher than air temperature near the land surface, this heat is released from the soil to the atmosphere as sensible heat, since latent heat flux is constrained by soil moisture deficits. If surface soil temperature is warmer than subsoil temperatures, this temperature can also be propagated through the soil, dissipating heat by conduction and increasing ground heat flux. The fraction of net radiation that is released in the form of sensible heat into the atmosphere due to the restriction on latent heat flux, will then increase air temperatures. This sensible heat will affect local and/or remote temperatures depending on atmospheric circulation^9^. The increase in air temperatures leads to vapour pressure deficits, which will increase evaporative demand from already dry soils, thus possibly further leading to decreased soil water content and more energy available to warm up soils (Fig. 2). Therefore, soil temperature acts as a key factor in the soil moisture–temperature feedback that may reinforce hot spells in the lower atmosphere due to the restrictions on soil moisture and evaporation during extremes. This is the case, for example, at the DE-Tha station located in eastern Germany, where multi-year changes in soil moisture and latent heat flux are restricted during the hottest week of each year, while sensible heat flux and TX7d trends based on air and soil temperatures are significantly increasing during hot extremes in the past decades (Extended Data Fig. 7). The ERA5Land data also support the link between soil temperatures, soil moisture and the evolution of hot extremes above the surface, showing a strong inverse correlation between soil moisture and soil temperature and a strong relationship between air and soil temperatures during TX7d extremes above the surface (Extended Data Fig. 8). Thus, drying areas under warm conditions could lead to more hot days when daily maximum soil temperatures are higher than daily maximum air temperatures, and there is a release of heat from the soil into the atmosphere.

## The role of soil temperatures in a warming climate

When the soil surface is warmer than the air above the surface, there is a heat exchange from the soil to the lower atmosphere in the form of sensible heat. This release of heat can contribute to the intensification and spreading of air hot extremes and heatwaves^28^. To investigate the possible contribution of soil temperatures to hot extremes near the surface in the future, the percentage of days with daily maximum soil temperatures higher than daily maximum air temperatures during air hot extremes were estimated. That is, the percentage of days when soil is releasing energy into the atmosphere during air hot extremes, not just during night but also at peak temperatures. The historical and SSP5-8.5 experiments from five CMIP6 models were used to study the change in the probability of occurrence of these events. Although there are large differences between climate models in simulating future conditions, particularly when soil processes are involved^7^, we study the probability of these events, being consistent with the physics of each individual model. Then, we investigate the agreement among models in the effect of climate change on the probability of events when soil temperatures are reinforcing air hot extremes.

**Fig. 3:**
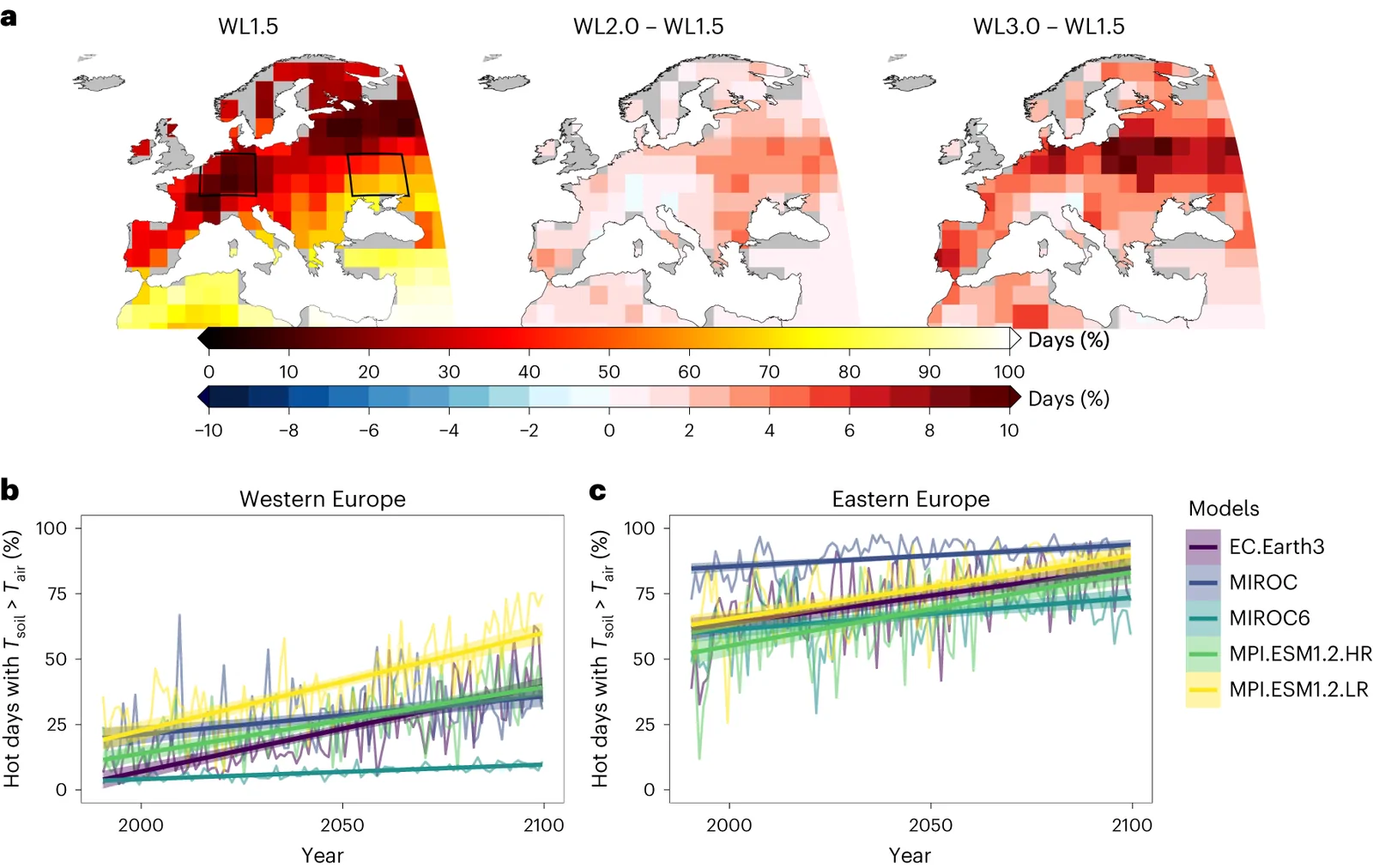
Percentage of days with a release of heat from soil into the atmosphere in summer. **a**, Percentage of days with maximum soil temperatures (*T*_Soil_) higher than maximum air temperatures (*T*_Air_) during air hot extremes as represented by the multimodel mean of the CMIP6 models under the 1.5 °C warming level and its difference with warming levels of 2.0 °C and 3.0 °C. Air hot extremes are defined on the basis of the TX90p index. **b**,**c**, Averaged percentage of days with *T*_Soil_ > *T*_Air_ over central-western (**b**) and central-eastern (**c**) Europe (see rectangles in **a**) from 1990 to 2100 for each model separately.

**Extended Data Fig. 9:**
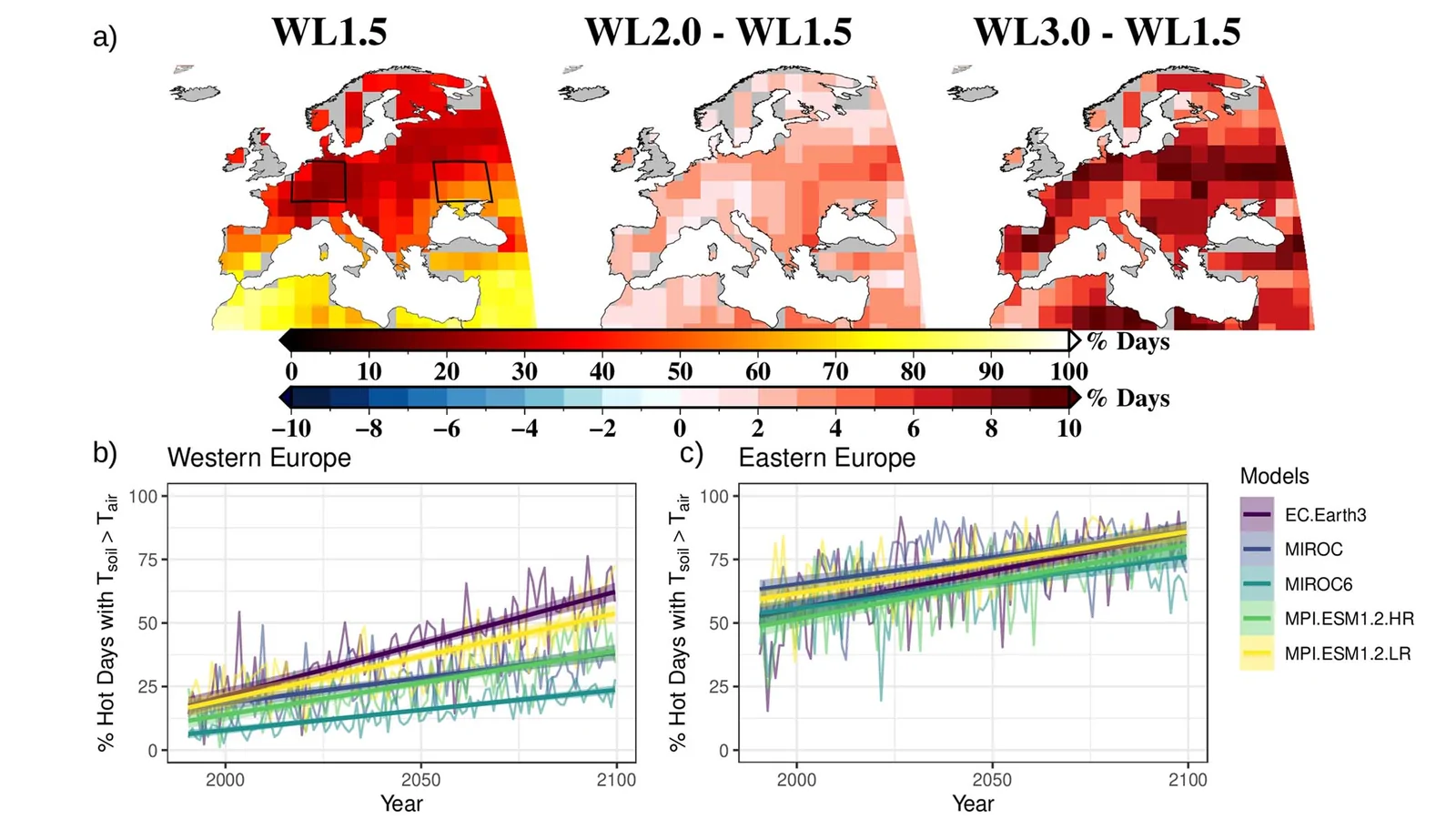
Percentage of days with a release of heat from soil into the atmosphere in summer. a) Percentage of days with daily mean soil temperatures (T_Soil_) higher than daily mean air temperatures (T_Air_) during air hot extremes as represented by the multimodel mean of the CMIP6 models under the 1.5 ^∘^C warming level and its difference with warming levels of 2.0 ^∘^C and 3.0 ^∘^C. Air hot extremes are defined based on the TX90p index. Averaged percentage of days with T_Soil_ > T_Air_ over central-western (b) and -eastern (c) Europe (see rectangles in a) from 1990 to 2100 for each model separately.

The CMIP6 models show a higher number of days with warmer soils than air during hot spells over Mediterranean areas and central-eastern Europe with an increasing trend over the whole Europe, during the twenty-first century (Fig. 3a). The changes in the percentage of days when soil temperature reinforces near-surface hot spells under the 2.0 °C and 3.0 °C warming levels in comparison with the 1.5 °C warming level, show larger changes in Central Europe, particularly large over eastern Europe, reaching regional differences between the 3.0 and 1.5 warming levels of more than 8% of hot days (Fig. 3). The spatial averages of the percentage of days when soil temperature reinforces hot spells over central-eastern and central-western Europe show some disagreement between models in the total number of days when these events happen. There is, however, unanimity among models in the increase in the probability of the occurrence of these events, although with different rates between models (Fig. 3). For example, in eastern Europe, all models indicate more than 10% increase in the days with a contribution of soil temperatures to hot spells at the end of the twenty-first century than in 1990 under the SSP5-8.5 emission scenario (Fig. 3b). In western Europe, four out of five models also indicate an increase of more than 10% in the days when soil temperature reinforces hot spells at the end of the twenty-first century (Fig. 3c). Similar conclusions are reached when comparing daily mean air and soil temperatures during hot spells above the surface, showing more consistency among models and larger trends, particularly in eastern Europe (Extended Data Fig. 9), which indicates the robustness of these results.

## Implications

Our results reveal that soil hot extremes are intensifying and becoming more frequent than air hot extremes at local and regional scales over Europe, particularly over Central Europe. This happens in areas during dry and warm conditions, where soil water content can not meet the requirements of evaporation and more energy is available to warm the soil. Meanwhile, atmospheric warming and the associated changes in precipitation over Europe could also lead to drier soils, contributing to the evolution of soil hot extremes.

The regional differences between air and soil hot extremes are of particular relevance for impact studies on agriculture and terrestrial ecosystem functions. Current impact and risk studies are usually based on surface air temperature observations (for example, ref. ^29^) due to the larger data availability above the land surface than within soil. In light of these results, studies on the impacts of hot extremes on agricultural activities and the terrestrial carbon budget based on air temperatures may underestimate the implications of the rapid increase in soil hot extremes. Thus, maximum soil temperatures should be included in impact and risk studies as a complementary perspective to the traditional approach.

Our findings further support the importance of the soil moisture–temperature feedback for the evolution of hot spells in a warming climate. Although all models indicate an increase in days when soil temperatures may reinforce hot spells via the soil moisture–temperature feedback, large differences between models were found. Thus, the representation of soil temperature and its role in the soil moisture–temperature feedback within climate models will have to be examined and improved to infer the exact contribution of soil temperatures to future hot extremes and heatwaves.

The fast increase in hot soil extremes is highly related to soil water loss, but other factors such as land cover change and land management could be affecting the different trends between air and soil hot extremes via changes in soil water content. Further research is required to understand all drivers and consequences of hot soil extremes. For example, extreme soil temperatures negatively impact the health of plants above and below the soil and increase soil carbon losses and soil water repellency. Further analyses are required to evaluate the consequences of the fast increase in soil hot extremes for the production of crop yields, threatening food security, and for biodiversity by favouring temperature-tolerant species. The fast increase in soil hot extremes could also curtail the efforts to increase soil carbon sequestration, revegetate degraded landscapes and limit desertification.

## Methods

### Datasets

Extreme indices are estimated on the basis of maximum air and soil temperatures from different data sources. Subdaily air and soil temperatures at depths shallower than 10 cm were obtained from the FLUXNET2015 dataset^16^, the ICOS^30–40^, the ARPAV^18^, DWD^19^ and Météo France^20^. Fluxnet and ICOS stations provide measurements every 30 min or 1 h, DWD and ARPAV stations provide data every hour and Météo France stations provide data every 6 h. We obtained instantaneous land surface skin temperatures for the period 1991–2015 from the CM SAF Land Surface Temperature dataset from Meteosat First and Second Generation Edition 1 (ref. ^21^) and maximum air temperatures from the E-OBS daily gridded land-only observational dataset^24^. From the hourly product of the ERA5Land re-analysis^22^, we used air temperatures defined at 2 m of height and soil temperatures and moisture at the first soil layer (0–7 cm) from 1996 to 2021. All subdaily temperature series were converted to daily resolution selecting the maximum temperature of each day. Only days with no missing values were used to produce the daily maximum temperatures; otherwise that day is considered as a missing value.

Other variables from the ICOS dataset were employed for the analysis of trends in surface fluxes and conditions during air hot extremes (Extended Data Fig. 7). For this analysis, we selected the ICOS database because it provides long-term measurements of atmospheric conditions and surface fluxes. However, each station has different conditions, and only 3 out of 11 ICOS stations show significant trends in the TX7d index based on air and soil temperatures during the hottest week of the year based on air temperatures (DE-Tha, BE-Vie and DK-Sor). And among these three stations, only DE-Tha is showing a faster increase in TX7d based on soil temperatures than in TX7d based on air temperatures during hot atmospheric conditions. The lack of flux measurements in other databases prevents us from extending this analysis to other sites showing faster increase in soil hot extremes than in air hot extremes.

### Extreme indices

Two definitions of hot extreme indices have been used in this study: (1) TX7d defined as the mean value of daily maximum temperatures over the hottest week per year and relevant as a measure of the intensity of hot extremes; (2) TX90p relevant for the frequency of hot extremes and defined as the percentage of hot days per month in summer. We considered hot days as the days when maximum temperature is above the calendar day 90th percentile centred on a 5 day window for the base period, defined in this study as the first 10 years of the dataset. The TX7d index was estimated for each year from all databases at stations or pixels with no more than 20 consecutive missing values from 1 April to 30 September. Other stricter criteria for managing missing values were tested, reaching similar results and conclusions (Extended Data Fig. 4). Meanwhile, the monthly TX90p index was estimated in months that include less than 10 days as missing values. Similar results are obtained when estimating the TX90p index only at months including less than 5 days as missing values (Extended Data Fig. 5). For the analysis of trends, the annual values of the TX90p index were estimated as the average of the monthly TX90p index in boreal summer (June, July and August). The use of the TX90p index during boreal summer allows the analysis of trends during the hottest and driest months of the year, when soil moisture feedbacks have a stronger impact on atmospheric conditions.

The indices were computed for the period 1996–2021 in order to use as many meteorological observations as possible. The analysis of ERA5Land outputs for a longer period (1970–2021) yields similar conclusions (Extended Data Fig. 2), which indicates that the length of the selected period is not affecting the conclusions of this study. Thus, the base period used for the TX90p index corresponds to 1996–2005 for the index built on all gridded products, but for each station the base period will change according to the data availability. Then, trends were estimated only at stations and pixels with more than 10 years of data. Trend significance and magnitude in the two extreme indices for all datasets were computed on the basis of the Mann–Kendall trend test and the non-parametric Sen’s slope estimator to reduce the effect of out-layers on our results. Significances were calculated at the 90% confidence level for all datasets and only stations presenting statistically significant trends in at least one of the index based on air or soil temperatures were included in the analysis. Thus, TX7d trends were estimated at a total of 118 stations (6 FLUXNET, 11 ICOS, 14 ARPAV, 40 DWD and 47 Météo France) corresponding with 160 pairs of air and soil measurements. Due to the different criteria used for estimating the indices, the number of stations at which TX90p trends were estimated is slightly different, being a total of 103 stations (11 ICOS, 14 ARPAV, 49 DWD and 29 Météo France) corresponding with 154 pairs of air and soil measurements. Since the distribution of our estimates over Europe is not homogeneous (Extended Data Table 1), spatial averages are driven by results from Germany, France and Italy, which are regions with a higher density of stations.

The comparison between trends in soil and air hot extremes was represented to answer two different questions. Column 3 in Fig. 1 is the difference between the absolute values of trends in soil and in air, which indicates where hot extremes are changing faster. In contrast, column 4 in Fig. 1 shows the difference between soil and air trends only when trends in soil and air are positive; thus, these maps show which hot extreme increases faster.

### Soil moisture–temperature feedback in a warming climate

Outputs of daily maximum air temperature (2 m) and 6 h soil temperature at 5 cm were obtained from the CMIP6-ESGF archive. Temperature outputs from the historical and SSP5-8.5 simulations were used for each model using the first realization. The number of models included in the analysis based on soil temperatures is limited to five (MIROC-ES2L (ref. ^41^), MIROC6 (ref. ^42^), MPI-ESM2-LR (ref. ^43^), MPI-ESM2-MR (ref. ^44^) and EC-Earth3 (ref. ^45^)), since only these models provide outputs of subdaily soil temperatures for the two simulations and they were required to estimate daily maximum soil temperatures. The study of soil temperature as heat contributor to future hot days developed near the surface was performed comparing daily maximum soil and air temperatures during hot days above the surface in summer (June, July and August). Hot days were identified using the monthly TX90p index based on air temperatures. Thus, we estimated the percentage of hot days when maximum soil temperatures are higher than maximum air temperatures. The same analysis was done comparing mean daily soil and air temperatures, reaching similar conclusions but higher percentages with mean temperatures than those using maximum temperatures (Extended Data Fig. 9). Results are presented using the multimodel mean, interpolating each final model result to the coarsest grid (MIROC-ES2L). To avoid the effect of the different climate sensitivity of each climate model on the results, we used three different warming levels (1.5, 2.0 and 3.0). Warming levels are estimated for each model realization as suggested by ref. ^46^.

## Online content

Any methods, additional references, Nature Portfolio reporting summaries, source data, extended data, supplementary information, acknowledgements, peer review information; details of author contributions and competing interests; and statements of data and code availability are available at https://doi.org/10.1038/s41558-023-01812-3.

## Supplementary information

## Figures and Tables

**Extended Data Table 1:**
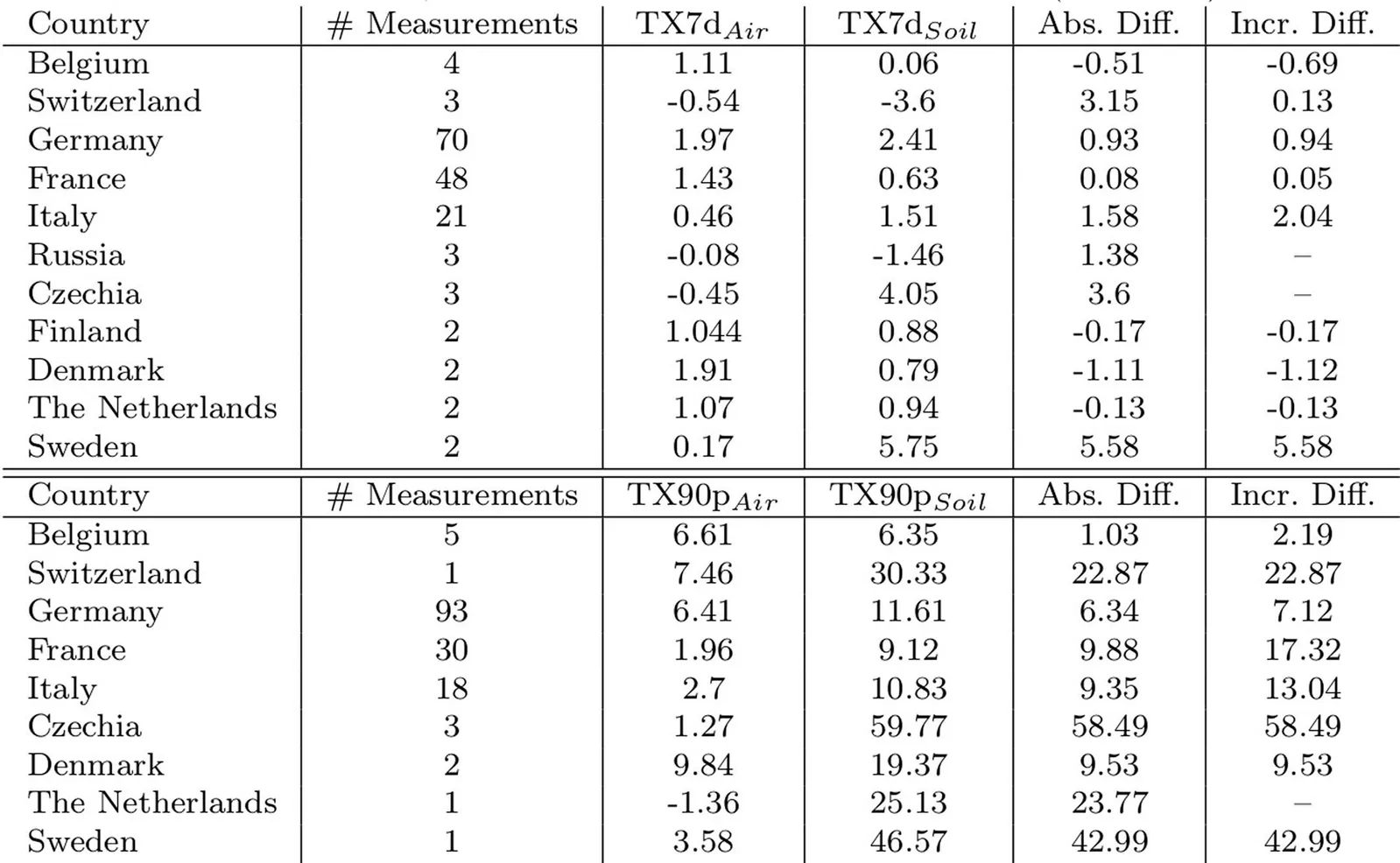
Summary of trends in TX7d and TX90p indices based on air and soil temperatures, grouped by country

The columns correspond with the country of the data series, the number of measurements (pairs of soil and air temperatures) in each country, the mean of trends in TX7d_Air_ and TX7d_Soil_, the differences between the absolute values of TX7d_Soil_ and TX7d_Air_ (Abs. Diff.) and the same difference only when trends in soil and air are positive (Incr. Diff.).

## Data Availability

In situ measurements were obtained from the FLUXNET2015 dataset^16^, the ICOS^30–40^, the ARPAV^18^, DWD^19^ and Météo France^20^. The CM SAF Land Surface Temperature dataset is available from https://www.cmsaf.eu/. The E-OBS and ERA5Land variables can be downloaded from the Copernicus Climate Service (https://climate.copernicus.eu/climate-reanalysis). The climate model outputs can be downloaded from the WCRP Coupled Model Intercomparison Project (Phase 6) https://esgf-data.dkrz.de/search/cmip6-dkrz/. All databases were provided by the indicated institutions through their webpages or on request. The observational data employed in this article will be shared on request with the consent from the corresponding agency.

## Code availability

The TX90 index and thresholds were produced using climdex.pcic in R and are available at https://CRAN.R-project.org/package=climdex.pcic. The Thiel–Sen slopes and the Mann–Kendall trend test were estimated using the R zyp and Kendall packages available at https://search.r-project.org/CRAN/refmans/zyp/html/zyp.htmland https://cran.r-project.org/web/packages/Kendall/index.html.
