# Supplementary material for: Soil heat extremes can outpace air temperature extremes
Source: Nat Clim Chang. 2023 Sep 21;13(11):1237–41. doi: 10.1038/s41558-023-01812-3 (PMC13538066; doi:10.1038/s41558-023-01812-3)
Supplement: Supplementary file 1 [file 41558_2023_1812_MOESM1_ESM.pdf]

nature climate change

Article

https://doi.org/10.1038/s41558-023-01812-3

# Soil heat extremes can outpace air temperature extremes

In the format provided by the authors and unedited

# Supporting Information

## Effect of soil depth on soil hot extremes

Regarding the effect of soil depth on trends in soil hot extremes, all soil temperatures included in this analysis correspond to temperatures of the top 10 cm of the soil. There are, however, some differences between datasets. FLUXNET, ICOS and DWD datasets include soil temperatures at 2, 5 and 10 cm, while ARPAV and Météo France provides soil temperatures only at 10 cm. These databases, except for the Météo France database, also provide soil temperatures at deeper soil layers, but the number of stations with deep measurements is largely reduced. For this reason, we decided to focus this analysis on the shallowest 10 cm of the soil.

A comparison of the probability distribution of trends classified by soil layer indicates higher trends in the soil than in the air at the three different layers (Extended Data Fig. 9). However, due to the different number of stations providing data at each soil layer and the high spatial variability of our results, we cannot reach any conclusion from this analysis. Due to the nature of heat propagation through the soil, we expect that the signal of daily maximum temperatures is attenuated at deeper layers. But further analyses focusing on a smaller selection of locations with a complete monitoring of the soil temperature profile would be necessary to investigate what is happening in the first meter of the soil. This type of analysis could be used to investigate the effect of soil hot temperatures on the health and growth of vegetation.
